# Sorghum Grain Polyphenolic Extracts Demonstrate Neuroprotective Effects Related to Alzheimer’s Disease in Cellular Assays

**DOI:** 10.3390/foods13111716

**Published:** 2024-05-30

**Authors:** Nasim Rezaee, Eugene Hone, Hamid R. Sohrabi, Stuart Johnson, Leizhou Zhong, Prakhar Chatur, Stuart Gunzburg, Ralph N. Martins, W. M. A. D. Binosha Fernando

**Affiliations:** 1 Centre of Excellence for Alzheimer’s Disease Research & Care, School of Medical and Health Sciences, Edith Cowan University, Joondalup, WA 6027, Australiae.hone@ecu.edu.au (E.H.);; 2 Department of Biomedical Sciences, Macquarie University, Sydney, NSW 2109, Australia; 3 Centre for Healthy Ageing, Health Futures Institute, Murdoch University, Murdoch, WA 6150, Australia; 4 School of Molecular and Life Sciences, Faculty of Science and Engineering, Curtin University, Perth, WA 6845, Australia; 5 CWEK PTY Ltd., Perth, WA 6845, Australia

**Keywords:** Alzheimer’s disease, antioxidants, neuroprotection, polyphenols, sorghum, amyloid-β, reactive oxygen species, mitochondrial function

## Abstract

Sorghum grain contains high levels and a diverse profile of polyphenols (PPs), which are antioxidants known to reduce oxidative stress when consumed in the diet. Oxidative stress leading to amyloid-β (Aβ) aggregation, neurotoxicity, and mitochondrial dysfunction is implicated in the pathogenesis of Alzheimer’s disease (AD). Thus, PPs have gained attention as possible therapeutic agents for combating AD. This study aimed to (a) quantify the phenolic compounds (PP) and antioxidant capacities in extracts from six different varieties of sorghum grain and (b) investigate whether these PP extracts exhibit any protective effects on human neuroblastoma (BE(2)-M17) cells against Aβ- and tau-induced toxicity, Aβ aggregation, mitochondrial dysfunction, and reactive oxygen species (ROS) induced by Aβ and tert-butyl hydroperoxide (TBHP). PP and antioxidant capacity were quantified using chemical assays. Aβ- and tau-induced toxicity was determined using the 3-(4,5-dimenthylthiazol-2-yl)-2,5-dimethyltetrazolium bromide (MTS) assay. The thioflavin T (Th-T) assay assessed anti-Aβ aggregation. The dichlorodihydrofluorescein diacetate (DCFDA) assay determined the levels of general ROS and the MitoSOX assay determined the levels of mitochondrial superoxide. Sorghum varieties Shawaya short black-1 and IS1311C possessed the highest levels of total phenolics, total flavonoids, and antioxidant capacity, and sorghum varieties differed significantly in their profile of individual PPs. All extracts significantly increased cell viability compared to the control (minus extract). Variety QL33 (at 2000 µg sorghum flour equivalents/mL) showed the strongest protective effect with a 28% reduction in Aβ-toxicity cell death. The extracts of all sorghum varieties significantly reduced Aβ aggregation. All extracts except that from variety B923296 demonstrated a significant (*p* ≤ 0.05) downregulation of Aβ-induced and TBHP-induced ROS and mitochondrial superoxide relative to the control (minus extract) in a dose- and variety-dependent manner. We have demonstrated for the first time that sorghum polyphenolic extracts show promising neuroprotective effects against AD, which indicates the potential of sorghum foods to exert a similar beneficial property in the human diet. However, further analysis in other cellular models and in vivo is needed to confirm these effects.

## 1. Introduction

Sorghum grain stands out as a promising functional food for health within human diets due to its unique qualities. Globally, it holds the fifth position among valuable cereal crops [1]. Some sorghum varieties, particularly those with outer bran layers, have higher levels of polyphenols (PPs) and antioxidant activity compared to other cereals [2]. Moreover, sorghum-derived foods retain notably elevated levels of polyphenolics and antioxidant capacity, attributed to the relative stability of polyphenols (PPs). This characteristic is especially noteworthy when compared to the polyphenols found in vegetables [3]. Additionally, research highlights the potential of sorghum PPs in mitigating chronic diseases like diabetes and cardiovascular conditions, both recognized as risk factors for Alzheimer’s disease [4]. Sorghum contains various types of polyphenols, including simple phenolic acids (such as ferulic and p-coumaric acids), 3-deoxyanthocyanidins, flavanones, flavones, other flavonoids, and condensed tannins [1]. Sorghum grains also contain rare compounds like luteolinidin and apigeninidin, which offer vasoprotective and anti-inflammatory benefits. Among these polyphenols, caffeic acid, trans-resveratrol, quercetin, catechin, cinnamic acid, cyanidin, apigenin, and kaempferol, in their purified forms, have garnered attention for Alzheimer’s disease (AD) prevention and treatment [5,6]. However, the effects of sorghum polyphenol extracts with their complex polyphenol profiles have not been reported.

Despite the growing health, social, and economic burden of AD worldwide, there is still no effective treatment or prevention method for the disease. Current therapeutic approaches only reduce the symptoms but are unable to stop or prevent the disease without adverse effects [7]. As such, there is an increasing interest in natural candidates considering their potentially lower side effects compared to synthetic drugs. Numerous scientific inquiries have shown the effectiveness of natural polyphenolic extracts in mitigating the neurotoxic impact of amyloid-β (Aβ), a hallmark of Alzheimer’s disease (AD). Extensive research indicates that both the oligomerized and fibrillar forms of Aβ are central to the progression of AD, exerting detrimental effects through interactions with various cellular processes. These interactions lead to neurotoxicity, oxidative damage, and inflammatory responses [8].

Although the exact mechanisms for mitigating amyloid-beta (Aβ) toxicity remain unclear, studies indicate that antioxidant pathways play a crucial role in combating Aβ-induced neurotoxicity [9,10]. Aβ-induced neurotoxicity stems from oxidative stress, marked by a significant rise in reactive oxygen species (ROS) levels as aging and Alzheimer’s disease advance. These ROS are primarily generated from the mitochondria and microglia [11,12]. Superoxide anion radicals, produced during mitochondrial electron transport chain activity, are pivotal in causing cellular oxidative stress. These highly reactive molecules, resulting from electron leakage, substantially damage cellular components and trigger the generation of additional reactive oxygen species (ROS), which affect cell signaling and redox balance regulation. Their generation within the mitochondria also influences cellular respiration, emphasizing the complex interplay between ROS production and mitochondrial function, thereby impacting cellular metabolism and the management of oxidative stress [13]. Antioxidants help to neutralize reactive oxygen species (ROS) and prevent oxidative damage. Therefore, antioxidant pathways present promising therapeutic strategies for mitigating the detrimental effects of Aβ accumulation in the brain due to oxidative stress. Further exploration of these pathways may pave the way for the development of innovative treatments for Alzheimer’s disease and other related conditions.

The literature shows that several phenolic-rich plant foods such as berries, spices, nuts, green tea, and olive oil have AD protective effects due to their antioxidant capacity [14]. The polyphenolic compound derived from the cat’s claw (*Uncaria tomentosa*) plant exhibits a notable ability to hinder the formation of aggregated Aβ fibrils or disaggregate preformed fibrils, mainly due to its proanthocyanidin (PP) components [15]. Similarly, a polyphenolic extract of green tea containing various PPs such as catechins and resveratrol has demonstrated reductions in Aβ plaque pathology in vitro and/or in vivo [16,17]. Additionally, the anti-Alzheimer’s disease (AD) effects of polyphenolic extracts from several dietary plants and their purified phenolic constituents, such as resveratrol (from grape and red wine) [18], anthocyanins (from berries) [19], and curcumin (from turmeric) [20] have been extensively documented. The flavonoid class of PP has demonstrated beneficial effects on Aβ aggregation and Aβ-induced reactive oxygen species (ROS) [21], whereas proanthocyanidins led to the disassembly of Aβ fibrils and tau-protein filaments and thus, the inhibition of plaque and tangle aggregation. The main proposed mechanism for this activity is their specific structure, namely the possession of adjacent hydroxyl groups that are attached to aromatic rings. Various polyphenols (PPs) present in sorghum have been studied individually for their potential protective effects against Alzheimer’s disease (AD). Compounds like apigenin, luteolin, and quercetin, found in sorghum, have been explored and demonstrated to inhibit the aggregation of amyloid-beta (Aβ) or to disaggregate Aβ fibrils [22].

The assessment of synergistic effects from natural drug combinations has emerged as a prominent area of investigation in phytomedicine research [23]. Pereira et al. suggest that these synergies within plant combinations could amplify their therapeutic benefits compared to individual compounds [24]. Consequently, the intricate combinations of polyphenols (PPs) present in sorghum grain extracts may exhibit similar synergistic effects on Alzheimer’s disease (AD) pathogenesis. Sorghum grains are emerging as a promising dietary component for Alzheimer’s disease (AD) due to their rich content of polyphenolic compounds, such as flavonoids and phenolics, which possess potent antioxidant and neuroprotective properties. These compounds have been shown to mitigate various pathological processes associated with AD, including the aggregation of amyloid-beta (Aβ) peptides, oxidative stress, and neuronal cell death. Research suggests that PP found in sorghum can enhance cell viability and reduce Aβ toxicity and aggregation in cellular models of AD. Furthermore, these PP have demonstrated the ability to decrease the levels of reactive oxygen species (ROS) and mitochondrial superoxide induced by Aβ, which are key contributors to neuronal damage in AD. Despite the abundance and diversity of polyphenols in sorghum grain, research into their potential protective effects against AD remains understudied and warrants greater attention [25].

In the current work, we, for the first time, have investigated the protective effect of PP-rich extracts from six different varieties of sorghum grain on Aβ-induced neurotoxicity in human neuroblastoma BE (2)-M17 cells. For this purpose, we used two different PP extraction solvents to find the more effective matrix to reduce Aβ-induced cytotoxicity and the associated cell death. In addition, the thioflavin T (Th-T) assay was performed to investigate whether these sorghum extracts can inhibit Aβ42 aggregation both for single extracts and their combinations. Then, the effects of the extracts on intracellular Aβ/TBHP-induced ROS and mitochondrial superoxide levels in the BE (2)-M17 cells using the 2′-7′-dichlorodihydrofluorescein diacetate (DCFDA) and MitoSOX assays were determined.

## 2. Materials and Methods

### 2.1. Materials

Formic acid, sodium carbonate, sodium hydroxide, sodium nitrite, aluminum chloride, ethanol, acetonitrile, Folin–Ciocalteau reagent, catechin, gallic acid, ferulic acid, caffeic acid, luteolin, apigenin, naringenin, and 1,1,1,3,3,3-hexafluoro-2-propanol (HFIP) trypsin (sequencing grade) and dimethyl sulfoxide (DMSO) were obtained from Sigma-Aldrich (St. Louis, MO, USA). Taxifolin, luteolinidin chloride, and apigeninidin chloride were purchased from Extrasynthese (Neuville-sur-Saône, France). Aβ-42 peptides were purchased from ERI Amyloid Laboratory, LLC (Oxford, MS, USA). 3-(4,5-dimenthylthiazol-2-yl)-2,5-dimethyltetrazolium bromide (MTS) was obtained from Abcam (Cambridge, MA, USA). Human neuroblastoma BE (2)-M17 cells were obtained from the American Type Culture Collection (ATCC, Manassas, VA, USA) (CRL-2267). Dulbecco’s modified Eagle’s medium–Ham’s nutrient mixture F-12 (DMEM/F12), fetal bovine serum (FBS) Hank’s balanced salt solution with calcium and magnesium (HBSS/Ca/Mg), and GlutaMAX™ supplement were obtained from Gibco, Thermofisher Scientific (New York, NY, USA). The DCFDA kit was obtained from ABCAM (Melbourne, Australia: ab113851) and the MitoSOX kit was purchased from Thermo Fisher (Waltham, MA, USA).

### 2.2. Methods

#### 2.2.1. Extraction of Phenolic Compounds

Whole grains of six varieties of sorghum namely black pericarp ‘Shawaya short black-1’, brown pericarp ‘IS131C’, white pericarp ‘QL12’, and three red pericarp ‘QL33/QL36’, ‘B923296’, and ‘QL33’ were chosen for their potentially diverse phenolic profiles and levels. The grains (except Shawaya short black-1) were grown at the Controlled Environment Facility, Queensland Bioscience Precinct, The University of Queensland (Brisbane, Australia) and provided to Curtin University (Bentley, Western Australia). Shawaya short-black 1 was grown at Curtin University. All the grains were air-dried, manually cleaned, vacuum packed, and then kept at −20 °C until the milling process. The whole grain samples were ground in a mill (CEMPTEC 1092, Foss Tecator, Höganäs, Sweden) so that the flour could pass through 100% through a 500 µm sieve. The resulting flours were kept in air- and moisture-proof packaging at −20 °C until the extraction process [26]. The extraction method was adapted from that of Svensson, Sekwati-Monang, Lutz, Schieber, and Gänzle [27] as optimized for sorghum [26]. Briefly, 10 mL of 60% food-grade ethanol was mixed with 1 g of the sorghum flour and the mixture was shaken in a water bath at 60 °C for 3 h. Then, supernatants were collected after centrifuging at 3220× *g* for 10 min at 4 °C. The pellets were resuspended and centrifuged in fresh 60% food-grade ethanol a further two times and the supernatants were collected and combined. The extracts were stored at −20 °C under nitrogen gas. The samples were extracted in duplicate.

#### 2.2.2. Determination of Total Phenolic Content

The phenolic content was measured using a modified Folin–Ciocalteu method [28]. 100 μL of the phenolic extracts was reacted with 2.5 mL of 0.2 N Folin–Ciocalteau reagent for 3 min, and then 2 mL of saturated sodium carbonate solution (75 g/L) was added to the mixture. After 2 h incubation in the dark at room temperature, the absorbance was read at 765 nm using a UV-1800 spectrophotometer (Shimadzu, Canby, OR, USA). Standards were made from gallic acid (0–360 mg/L) and the results were expressed as mg gallic acid equivalents (GAEs)/g sorghum flour (dry basis, db). All the extracts were analyzed in duplicate.

#### 2.2.3. Determination of Flavonoid Content

The flavonoid content was measured using a modified method of Zhishen, Mengcheng, and Jianming [29]. 250 μL of extract was mixed with 75 μL of 5% NaNO_2_ and 1 mL of deionized water. After 5 min incubation at room temperature, 75 μL of 10% AlCl_3_ was added. Then, after 6 min, 500 μL of 1 mol/L NaOH and 600 μL water were added. Immediately after that, the absorbance of the mixture was read at 510 nm. Catechin (20–100 mg/L) was used as a standard. The results were expressed as mg catechin equivalents (CEs)/g sorghum flour (db). All the extracts were analyzed in duplicate.

#### 2.2.4. Determination of Profile and Level of Individual Phenolic Compounds

The PP profile of the extracts and the level of individual PP was determined using an Agilent 1100 HPLC coupled with a diode array detector (DAD) (Agilent Technologies, Palo Alto, CA, USA) [30]. A Kinetex XB-C 18 reversed phase-HPLC column (5 µm, 250 × 4.6 mm, Phenomenex, Torrance, CA, USA) was used. The signals between 190 and 600 nm at steps of 2 nm were scanned by the DAD. Solvents A and B were formic acid (0.1%) and acetonitrile (0.1%), respectively. Linear gradient elution was used as follows: 5–15% B (5 min); 15–50% B (40 min); 50–70% B (2 min); 70–100% B (1 min); 100% B (7 min); 100%-5% B (1 min); 5% B (9 min). The injection volume was 5 μL and the flow rate was 0.5 mL/min [30]. The following individual standards in 60% ethanol were used: ferulic acid, caffeic acid, luteolinidin, apigeninidin, luteolin, apigenin, trans-cinnamic, taxifolin, and naringenin. The identification of the sample peaks was performed by comparing the retention time and absorption spectra of the standards. Quantitation was by peak area. The results were expressed as µg/g sorghum flour (db). All the extracts were analyzed in duplicate.

#### 2.2.5. Chemical-Based Antioxidant Assays

The determination of the total antioxidant activity of the sorghum varieties was carried out using free radical-scavenging methods based on those of based on previous work [31]. Both the 1,1-diphenyl-2-picrylhydrazyl (DPPH) and 3-ethylbenzothiazoline-6-sulfonic acid diammonium salt (ABTS) assays were used. A stock solution for the DPPH assay was prepared by dissolving 24 mg of DPPH in 100 mL of methanol. A DPPH working solution with an absorbance of 1.1 ± 0.02 units at 515 nm was prepared by diluting 10 mL of this stock with 50 mL of methanol. Then, 150 μL of the sorghum extracts was mixed with the 2850 μL working solution. After 8 h of reaction time, the absorbance was measured at 515 nm using an LED spectrophotometer (Shimadzu, Canby, OR, USA). The standard curve consisted of Trolox (20–250 mg/L).

For the ABTS assay, a working solution was prepared by reacting equal quantities of 7.4 mM ABTS and 2.6 mM potassium persulphate for 12 h in the dark. Then, 150 µL of the phenolic extracts was added to 2850 μL of ABTS working solution. A UV-1800 spectrophotometer (Shimadzu, Canby, OR, USA) was used to measure the absorbance at 734 nm after 2 h of reaction time at room temperature. Both the DPPH and ABTS results are expressed as mg Trolox equivalents/g sorghum flour (dry basis). All the extracts were analyzed in duplicate.

#### 2.2.6. Preparation of the Sorghum Extracts for Cell Treatment

The extracts were dried using a SpeedVac (Thermo Scientific, SPD131DDA, Waltham, MA, USA). Then, half of the residue was resuspended in DMSO and the other half resuspended in treatment media (DMEM/F12 supplemented with 1% (*v*/*v*) FBS) to compare the effect of the resuspension solvent and stored at −80 °C.

#### 2.2.7. Aβ Oligomer Preparation

The Aβ-42 oligomers were prepared based on the method described by Stine et al. [32]. Briefly, the synthetic Aβ42 was dissolved in hexafluoro-2-propanol (HFIP) to form a stock of 1 mM. The aliquots of this Aβ42 stock were air-dried overnight forming films in microtubes. The residual solvent was removed using a SpeedVac (Thermo Scientific, SPD131DDA) and then the films were stored at −20 °C with desiccant until required. For the experiments, the Aβ films were resuspended in anhydrous DMSO at a concentration of 5 mM. The solution was then sonicated at room temperature for 10 min. Phenol red-free F12 media was added to a final concentration of 100 μM and incubated for 24 h at 4 °C protected from light before usage.

#### 2.2.8. Cell Culture

The human neuroblastoma BE (2)-M17 cells were cultured in DMEM/F12 supplemented with 10% (*v*/*v*) FBS and kept in a humidified incubator at 37 °C with 5% CO_2_/95% air. The cells were seeded into flasks (25 mL tissue culture flask with filter cap) and allowed to attach for at least 24 h such that they reached 80 to 90% confluency before the experiment.

#### 2.2.9. Cytotoxicity of Sorghum Extracts on BE (2)-M17 Cells

Approximately 1 × 10^4^ BE (2)-M17 cells were seeded per well in a 96-well plate and allowed to attach for 24 h before the treatment day. To find the non-toxic and optimum PP dosage of each extract (different sorghum varieties/different solvents), nine different PP dosages (4000, 2000, 1000, 750, 500, 250, 100, 10, and 1 µg sorghum flour equivalents/mL) of the extracts were prepared and checked through a cell viability assay (MTS). Briefly, the cells were treated with the resolubilized PP extracts for 72 h before the culture media were removed and replaced with 20 µL of the MTS reagent + 80 µL of the treatment media. After that, the samples were incubated for 4 h at 37 °C in the dark. A microplate reader (PerkinElmer, EnSpire multimode plate reader, Waltham, MA, USA) was used to determine the absorbance at 450 nm at room temperature. The optimum dosage (which was the highest non-toxic dosage that also does not have a high growth effect) and one lower dosage (1/2 of the optimum dosage) were selected for further experiments.

The maximum non-toxic concentration of DMSO in the extracts was also determined using MTS. According to our experiments for any concentration higher than 1.5% DMSO (*v*/*v*) in the treatment media, DMSO was significantly toxic to the cells compared to the control group (the cells treated with the treatment media only). Before the cell assays, the extracts were diluted in the treatment media to achieve the non-toxic concentration of DMSO and the non-toxic concentration of the PP extract for each sorghum variety.

#### 2.2.10. Cytoprotectively of Sorghum Extracts

The MTS assay was used to evaluate the protective activity of the sorghum extracts against the Aβ-induced cytotoxicity. For this purpose, the protectivity experiments were carried out with the selected dosage of each extract (see Section 2.2.9) and one lower dosage of each extract to check the dosage effect. The cell viability of the negative control group was considered 100%. From the preliminary results, 20 µM Aβ42 was selected for the positive control group to reach 40–50% cell death compared to the negative control group. Earlier experiments used the same concentration of Aβ42 [33,34]. The average relative cell viability of the positive control group was 58%. All the results were calculated based on the formula number (1), (2), and (3).

To assess the effect of sorghum extracts against Aβ-induced cytotoxicity, BE (2)-M17 cells were seeded at a density of 1 × 10^4^ cells per well in a 96-well plate for 24 h prior to the treatment. Then, the cell culture medium was removed and replaced with the following treatments:

- Negative control group (NC) (the cells treated with the treatment media and the equivalent amount of DMSO and phenol red-free f12, without Aβ42, and without extracts);
- Positive control group (PC) (the cells treated with 20 μM Aβ42, without extracts);
- Extract control group (EC) (the cells treated with the optimum dosage or ½ of the optimum dosage of each extract, without Aβ42);
- Extract-treated group (ET) (the cells treated with the optimum dosage or ½ of the optimum dosage of each extract + 20 μM Aβ42);
- Blank group (no cells, just the treatment media).

After 72 h incubation, the solutions in all the wells were removed, and then replaced with an equivalent volume of fresh treatment media supplemented with 20 μL of the MTS reagent (Abcam, Cambridge, MA). The plates were then incubated at 37 °C under CO_2_ for 4 h. Then, the absorbance was measured at 450 nm by a microplate reader (PerkinElmer, EnSpire multimode plate reader, USA) at room temperature. Each experiment was repeated four independent times.

The cell viability percentage was calculated by the following Equations (1)–(3) in which the negative control value was set to 100%:

Control cell viability (%) = [PC/NC] × 100(1)

Extracts cell viability (%) = [ET/EC] × 100(2)

Extracts rescue capacity (%) = Extract cell viability (%) − Control cell viability (%)(3)

#### 2.2.11. Thioflavin-T Aggregation Assay

For the thioflavin-T (Th-T) aggregation assay, frozen Aβ-42 peptide films were dissolved in 20 μL of DMSO, centrifuged for 1 min at 13,000× *g,* and then sonicated at 15–20 °C for 15 min in a sonicating water bath. Then, 980 μL of the ice-cold Ham’s F12 phenol red-free media was added to the peptide solution to a final concentration of 100 μM. The reagents were added to each well of the black–clear bottom 96-well plate in the following quantities:

- Positive control (20 μL Aβ-42 solution in DMSO/F12, 10 μL 60 µM Th-T solution, and 70 μL 1 × TBS).
- Negative control (20 μL DMSO/F-12 solvent only, 10 μL 60 µM Th-T solution, and 70 μL 1 × TBS).
- Extract control group (20 μL DMSO/F-12 solvent only, 10 μL 60 µM Th-T solution, and 70 μL of 1 × TBS/PP extracts in 1 × TBS).
- Combination group (20 μL of the prepared peptides, 10 μL of 60 µM Th-T solution, and 70 μL of 1 × TBS/extract dissolved in 1 × TBS).

The plate was then incubated for 23 h in a Perkin–Elmer plate reader and a fluorescence reading at the emission and excitation wavelengths of 490 and 450 nm, respectively, was made every hour. The experiment was repeated two times with five replicates of each group. Th-T fluorescence data were normalized to time 0 by the subtraction of the value (t = 0) of each group from each of the readings of that group.

#### 2.2.12. Cellular ROS Production Assay

Cellular ROS production was measured using a DCFDA assay kit (Abcam, Cambridge, MA, USA) according to the manufacturer’s protocol. The M17 neuroblastoma cells were seeded into black 96-well plates at a density of 25,000 cells per well. After 24 h, the cells were washed twice with 1x wash buffer (provided by the kit). Then, diluted DCFDA (25 μM) was added to the wells and the plate was placed back into the cell culture incubator at 37 °C for 45 min. After two washes with HBSS, the cells were exposed to the different treatment groups for 4 h:

- Blank group: no cells, 100 μL of just the extracts or treatment media (no phenol red DMEM/f12 + 1% FBS);
- Treatment groups: the cells treated with 30 μM Aβ + 70 μL of the extracts (at two non-toxic doses) and the cells treated with 50 μM TBHP diluted with the extracts (at two non-toxic doses);
- Negative control group: the cells treated with 70 μL of the treatment media + 30 μL of no phenol red or DMSO.
- Positive control groups: 30 μM Aβ + 70 μL treatment media and 50 μM TBHP diluted with the treatment media [34].

Previously, different concentrations of Aβ (20 μM, 25 μM, 30 μM, 35 μM, and 40 μM) were tested to find the optimized concentration which produces a high level of ROS. Among all the mentioned concentrations, 30 μM was the best in terms of high ROS generation. The fluorescence signal was measured at 488 nm excitation and 525 nm emission using a microplate reader. The relative changes in DCF fluorescence are expressed as fold increases over the fluorescence signal of the positive control group.

#### 2.2.13. Determination of Mitochondrial Superoxide Production in Live Cells

The mitochondrial-derived superoxide production of the cells was determined using a MitoSOX assay kit (Thermofisher Scientific) according to the manufacturer’s protocol [30]. Briefly, the neuroblastoma cells were seeded into black 96-well plates at a density of 12,500 cells per well. After 24 h, the cells were washed and treated for 4 h according to the following treatment groups:

- Blank group: no cells, 100 μL of the extracts or treatment media (no phenol red DMEM/f12 + 1% FBS);
- Treatment groups: the cells treated with 30 μM Aβ + 70 μL of the extracts (optimum dosage only) and the cells treated with 50 μM TBHP diluted with the extracts (optimum dosage only);
- Negative control group: the cells treated with 70 μL of the treatment media + 30 μL of no phenol red DMEM/f12;
- Positive control group: 30 μM Aβ + 70 μL treatment media and 50 μM TBHP diluted with the treatment media.

The cells were then washed twice with HBSS/Ca/Mg and stained with a 5 µM solution of MitoSOX in HBBS/Ca/Mg and incubated for 15–20 min at 37 °C in the dark. The stained cells were then washed twice with the warm buffer (provided by kit) and imaged using a fluorescence microscope (Eclipse, Ti2-E, Nikon, Tokyo, Japan). MitoSOX Red was detected using excitation and emission wavelengths at 510 nm and 580 nm, respectively. The images were quantified using the software (Fiji is just) ImageJ 1.53f51 (National Institutes of Health, Bethesda, MD, USA) to obtain the total integrated density values. The data were calculated and expressed as a percentage of the positive control group.

### 2.3. Statistical Analysis

The data presented in this study are represented as the mean value along with the standard deviation (SD), which indicates the variability within the dataset. The number of independent experiments conducted varied between two and four depending on the specific assay being performed. To determine the significance of the differences observed in the data, statistical analysis was conducted using a combination of the one-way analysis of variance (ANOVA) followed by a *t*-test. The significance level for determining statistical significance was set at *p* ≤ 0.05, indicating that the observed differences were considered statistically significant if the probability of their occurrence by random chance was less than or equal to 5%. This approach ensures robust statistical analysis and strengthens the reliability of the findings reported in the study.

## 3. Results

### 3.1. Polyphenolic and Flavonoid Contents of Sorghum Grain

The PP content of the six varieties of sorghum grain is presented in Table 1. The sorghum grain was found to have varying levels of PPs depending on the variety. For instance, Shawaya short black-1 and IS1311C exhibited notably higher (*p* ≤ 0.05) concentrations of PPs compared to other varieties. This outcome was anticipated due to their darker grain color, which is associated with elevated levels of PPs [26,35].

As expected, the flavonoid levels in the grain samples also vary according to variety as shown in Table 1. Compared to a previous study using the same varieties of sorghum [26], the level observed in the current study is lower for IS1311C but higher for QL33/QL36 to QL33. The highest flavonoid concentration (*p* ≤ 0.05) was observed in Shawaya short black-1 followed by IS1311C and QL33/QL36, while QL33, QL12, and B923296 had similar (*p* > 0.05) levels.

### 3.2. Identification and Quantitation of the Individual Polyphenolic Compounds of Sorghum Extracts

The retention times of the authentic PP standards were: luteolinidin (18 min), apigeninidin (21 min), luteolin (33), apigenin (38 min), taxifolin (24 min), naringenin (25 min), trans-cinnamic acid (36 min), ferulic acid (23 min), and caffeic acid (17 min). Both the presence/absence and amount of each PP were dependent on the sorghum variety (Table 2). All nine PPs were identified in Shawaya short black-1, seven in IS1311C and QL33, six in QL33/QL36, and only five B923296 and QL12, whilst Shawaya short black-1 was the only sample to contain luteolin and taxifolwiwn was only present in Shawaya short black-1 and IS1311C (Table 2). Shawaya short black-1 contained a higher level (*p* ≤ 0.05) of luteolinidin, apigeninidin, and luteolin than all the other varieties. Apigenin and ferulic acid were higher (*p* ≤ 0.05) in QL33. Naringenin was identified in all the sorghum varieties, except QL12. Moreover, trans-cinnamic acid was also identified in all the sorghum varieties, except IS1311C and B923296 (Table 2).

### 3.3. Free Radical-Scavenging Ability of the Extracts

The chemical-based DPPH and ABTS assays were used to assess the free radical-scavenging activity of the polyphenolic extracts of the six different varieties of sorghum grains. The results of these assays are presented in Table 3. The extracts of Shawaya short back-1 and IS1311C showed the highest (*p* ≤ 0.05) free radical-scavenging activity, with QL33/QL36, B923296, and QL12 exhibiting markedly lower (*p* ≤ 0.05) activity.

### 3.4. Cytotoxicity of Sorghum Extracts on BE(2)-M17 Cells

The extracts of all the sorghum grain varieties equivalent to 4000 µg sorghum flour equivalents/mL significantly reduced the viability of the BE(2)-M17 cells as measured by MTS where the viabilities of the extract-treated cells are normalized to the viability of the cells in the negative control group. However, at the lower concentrations equivalent to 2000 µg/mL and 1000 µg/mL, only the extracts of Shawaya short black-1 and IS1311C were cytotoxic. For the extracts of QL33/QL36 and B923296, the cells actually proliferated at a dosage of 2000 µg/mL. The concentrations below 750 µg/mL (ie. 500, 250, 100, 10, 1 µg/mL) were identified as non-cytotoxic for any of the extracts. For further cell studies, a concentration of 750 µg sorghum flour equivalents/mL was selected for the extracts of Shawaya short black-1 and IS1311C, 1000 µg/mL for QL33/QL36 and B923296, and 2000 µg/mL for QL12 and QL33.

### 3.5. Protective Effect of DMSO Sorghum Extracts on Aβ-Induced Cytotoxicity

**Figure 1:**
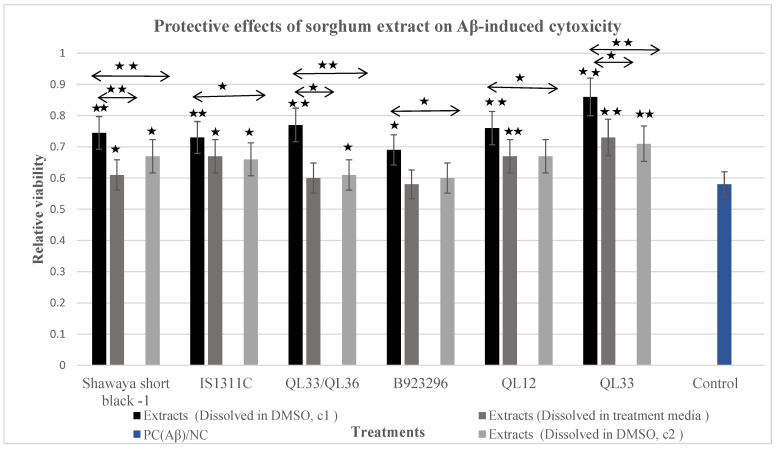
Protective effects of the six different varieties of sorghum extracts dissolved in two different solvents on the BE (2)-M17 cells against the Aβ-induced neurotoxicity of the BE (2)-M17 cells using the MTS assay. All the samples were compared to the negative control set at 100% viability. The concentrations C1 (C2) of the extract of each sorghum variety calculated in terms of the sorghum flour equivalents were Shawaya short black-1, 750 µg/mL (375 µg/mL); IS1311C, 750 µg/mL (375 µg/mL); QL33/QL36, 1000 µg/mL (500 µg/mL); B923296, 1000 µg/mL (500 µg/mL); QL12, 2000 µg/mL (1000 µg/mL); and QL33, 2000 µg/mL (1000 µg/mL). The half dosages were tested for the DMSO extracts only. The data are expressed as mean ± SD (*n* = three to four independent assays). The statistically significant differences in comparison to the control are marked (*) for (*p* ≤ 0.05) and marked (**) for (*p* ≤ 0.01).

The incubation of the BE (2)-M17 cells with A-β and the optimal dosage of the DMSO sorghum extracts resulted in significant protection as seen by an increased cell viability (*p* ≤ 0.05) for some treatment groups compared to the positive control (Figure 1). The DMSO extracts preserved cell viability as follows: Shawaya short black-1, 74%; IS1311C, 73%; QL33/QL36, 77%; B923296, 69%; QL12, 76%; and QL33, 86%. Thus, the rescue capacity of the extracts was as follows: Shawaya short black, 16%; IS1311C, 15%; QL33/QL36, 19%; B923296, 11%; QL12, 18%; and QL33, 28%. As expected, the protective effect of the half doses of each extract was weaker (*p* ≤ 0.05) than the optimum dosage (Figure 1). However, lower doses were significantly effective in Shawaya short black-1 (*p* ≤ 0.05), IS1311C (*p* ≤ 0.05), QL33/QL36 (*p* ≤ 0.05), and QL33 (*p* ≤ 0.01). In summary, the protective effects of the DMSO sorghum extracts were dose- and variety-dependent. The order of potency for preserving cell viability against Aβ -induced toxicity was QL33 > QL33/QL36 > QL12 > Shawaya short black-1 > IS1311C > B923296 based on the optimum dose for each variety.

### 3.6. Protective Effect of Treatment Media Sorghum Extracts on Aβ-Induced Cytotoxicity

The cell viability of the optimum dosages of the treatment media extracts was as follows: Shawaya short black-1, 61%; IS1311C, 67%; QL33/QL36, 60%; B923296, 58%; QL12, 67%; and QL33, 73% (Figure 1). Thus, the rescue capacity of the treatment media extracts was Shawaya short black-1, 3%; IS1311C, 9%; QL33/QL36, 2%; B923296, 0%; QL12, 9%; and QL33, 15%. The rescue capacity was highly significant (*p* ≤ 0.01) for Shawaya short black-1, IS1311C, and QL33 but not significant (*p* > 0.05) for the others. Among the sorghum varieties, QL33 and B923296 showed the highest and lowest effectiveness for these extracts (*p* ≤ 0.01). In addition, for all the sorghum varieties, the extracts dissolved in DMSO showed a higher rescue capacity than the extracts dissolved in the treatment media (*p* ≤ 0.05). For this reason, the optimum dosages of the extracts dissolved in DMSO were used for further experiments.

### 3.7. Anti-Aβ Aggregation Effect of Sorghum Extracts

**Figure 2:**
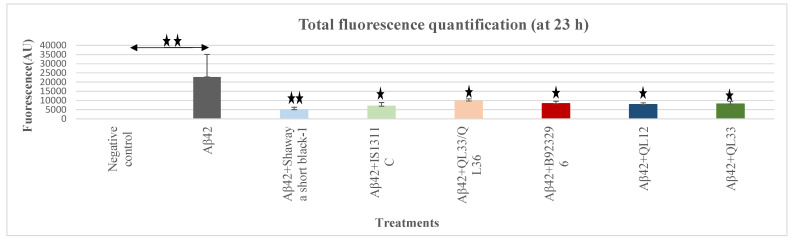
Total fluorescence quantification (23 h) as a measure of self-induced aggregation kinetics using the Thioflavin-T assay. The statistically significant differences are marked (*) for (*p* ≤ 0.05) and marked (**) for (*p* ≤ 0.01). Negative control = sorghum extracts only; Aβ42 = positive control (20 µM Aβ42 only); six different varieties were assessed over a 23 h period by the (Th-T) fluorescence assay. Th-T fluorescence is represented as arbitrary units (AUs).

A gradual increase in the self-induced aggregation of Aβ42 only (positive control) as measured by fluorescence was observed during the Th-T assay period. In contrast, for the negative control and extract control groups, no increase in aggregation trend was observed. For the combination of Aβ42 and sorghum extracts, the aggregation was significantly reduced compared to the positive control group (Aβ42 only) for the extracts of all sorghum varieties (Figure 2) and the aggregation steeply increased until t = 2 h and then plateaued till the end of the experiment at t = 23 h. The total fluorescence was quantified after 23 h incubation time to compare the aggregation levels at the endpoint of the experiment. The extracts of all the sorghum varieties significantly inhibited the Aβ42 aggregation (*p* ≤ 0.05) (Figure 2). The percentage of the total aggregation inhibition was as follows: Shawaya short black-1, 78%; IS1311C, 69%; QL33/QL36, 57%; B923296, 63%; QL12, 65%; and QL33, 64%. The sorghum extracts of Shawaya short black-1 and B923296 demonstrated the highest (*p* ≤ 0.05).

### 3.8. Effect of PP-Rich Sorghum Extracts on Aβ-Induced ROS in BE (2)-M17 Cells

**Figure 3:**
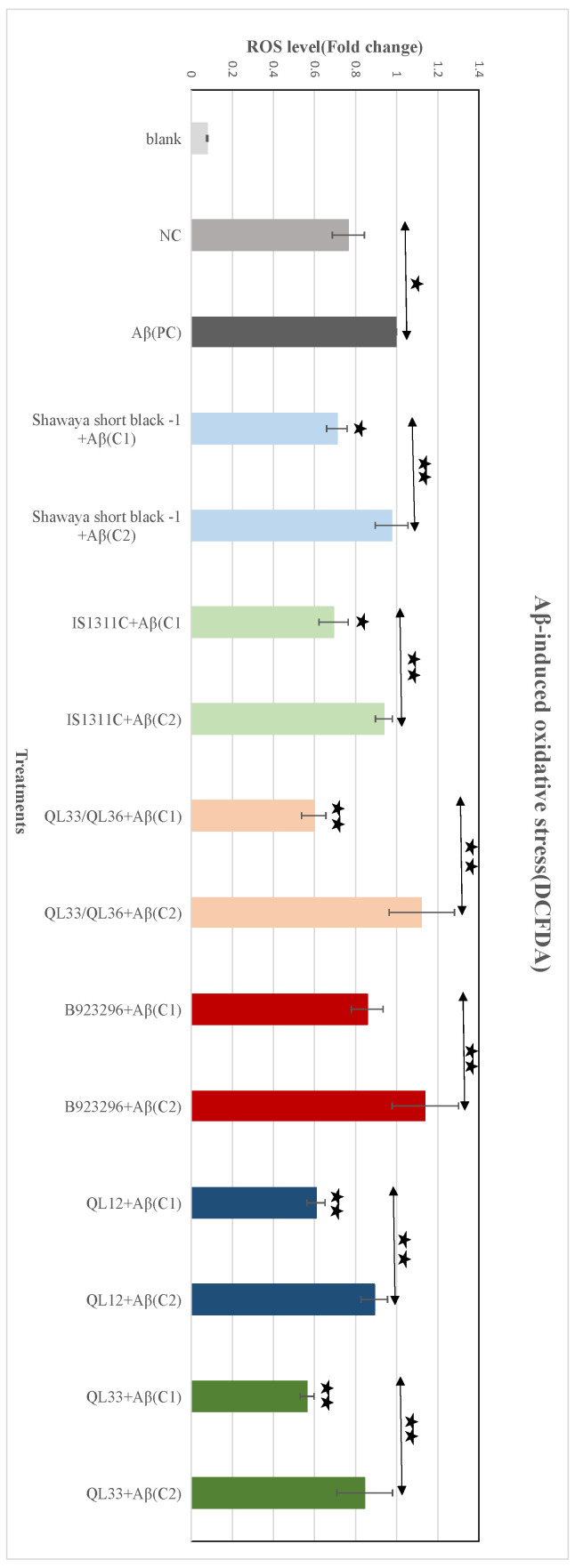
The effects of PP-rich extracts from the six different varieties of sorghum on the Aβ-induced oxidative stress in the BE (2)-M17 cells. The ROS level is expressed as a fold change in the treatments compared to the positive control. All the values are presented as mean ± SD of four independent experiments (*n* = 4). Each experiment included three replicates. The statistically significant differences in comparison to the positive control are marked (*) for (*p* ≤ 0.05) and marked (**) for (*p* ≤ 0.01). NC = negative control, PC = positive control.

The extracts of the six different varieties of sorghum grain were tested for their ability to attenuate intracellular Aβ-induced ROS using the DCFDA assay kit. Adding Aβ to the negative control group significantly increased ROS levels by 30.8% compared to the negative control group (*p* ≤ 0.05) (Figure 3). In comparison to the positive control, the extract of Shawaya short black-1 (750 µg/mL) reduced ROS by 29.1% (*p* ≤ 0.05), IS1311C (750 µg/mL) by 30.7% (*p* ≤ 0.05), QL33/QL36 (1000 µg/mL) by 40.3% (*p* ≤ 0.01), B923296 (1000 µg/mL) by 14.3%, Q12 (2000 µg/mL) by 39.2% (*p* ≤ 0.01), and QL33 (2000 µg/mL) by 43.6% (*p* ≤ 0.01). The B923296 (1000 µg/mL) treatment group was not significantly different from the positive control (*p* > 0.05). The lower (half) dosage of each extract was significantly weaker (*p* ≤ 0.05) in terms of the attenuation of Aβ-induced ROS compared to the optimum dosage. None of the lower dosages significantly reduced ROS compared to the positive control (Figure 1). In summary, the data presented in Figure 1 indicate that optimum dosages of Shawaya short black-1, IS1311C, QL33/QL36, QL12, and QL33 have significant antioxidative properties on Aβ-induced BE (2)-M17 cells.

### 3.9. Effect of PP-Rich Sorghum Extracts on TBHP-Induced ROS in BE (2)-M17 Cells

**Figure 4:**
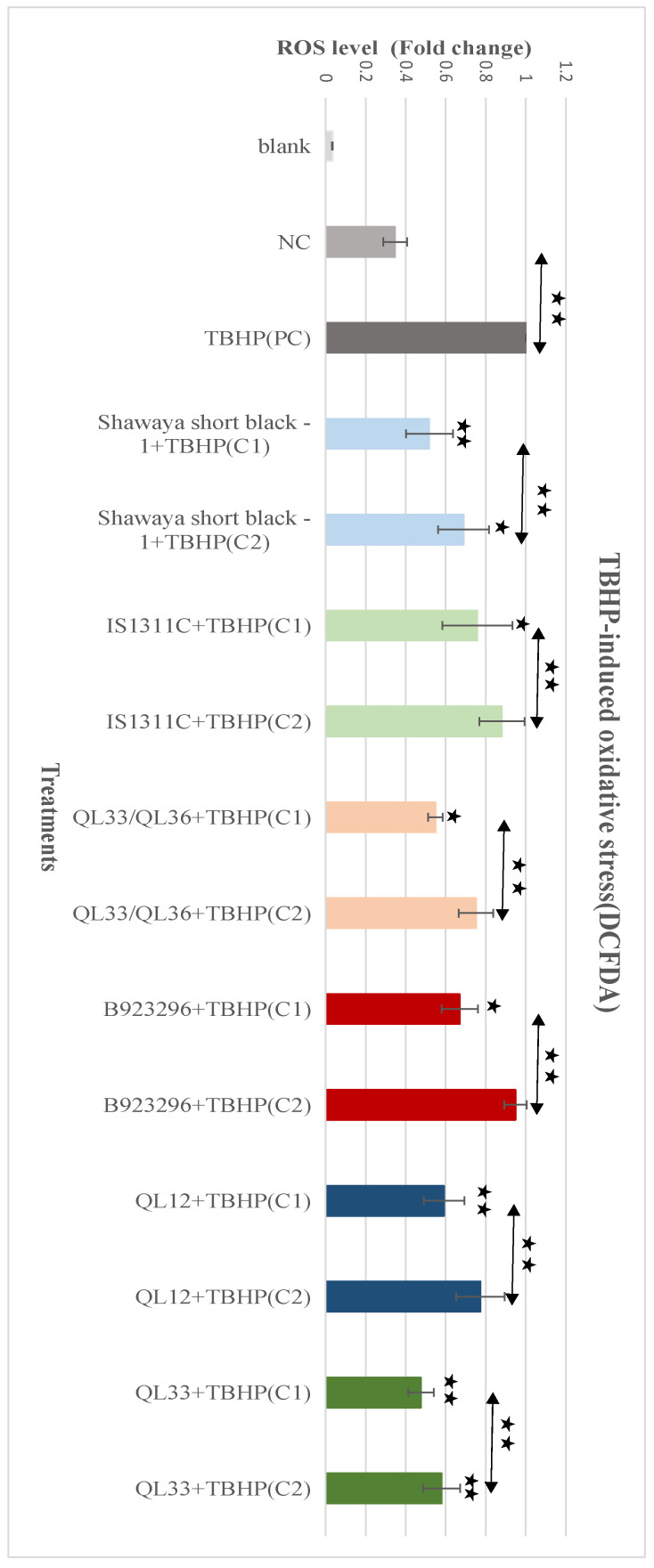
The neuroprotective effects of the PP-rich extracts from the six different varieties of sorghum on the TBHP-induced oxidative stress in the BE (2)-M17 cells. The initial cellular ROS level was obtained from a fluorescence microplate reader using a DCFDA kit. The ROS level is expressed as a fold change in PC after background subtraction. All the values presented correspond to mean ± SD of two to four independent experiments. Each experiment included two replicates. The statistically significant differences in comparison to the PC are marked (*) for (*p* ≤ 0.05) and marked (**) for (*p* ≤ 0.01). NC = negative control, PC = positive control.

The DCFDA assay was also used to measure the effects of the sorghum extracts on intracellular ROS induced by TBHP. Figure 2 shows that adding TBHP to the negative control group significantly increased ROS by 187.5%. A significant reduction in ROS levels was achieved by adding the sorghum extracts compared to the positive control, as shown in Figure 4. ROS attenuation was highly significant for the Shawaya short black-1 (750 µg/mL), QL12 (2000 µg/mL), and QL33 (2000 µg/mL) varieties (*p* ≤ 0.01), and significant for IS1311C (750 µg/mL), QL33/QL36 (1000 µg/mL), and B923296 (1000 µg/mL) (*p* ≤ 0.05). The lower (half) dosages of each extract showed a lower attenuation effect (*p* ≤ 0.05) compared to the optimum dosage (Figure 2); however, the lower dosages of Shawaya short black -1 (375 µg/mL) and QL33(1000 µg/mL) was still significantly effective (*p* ≤ 0.05) compared to the positive control.

### 3.10. Effect of PP-Rich Sorghum Extracts on Aβ-Induced Mitochondrial Superoxide in BE (2)-M17 Cells

**Figure 5:**
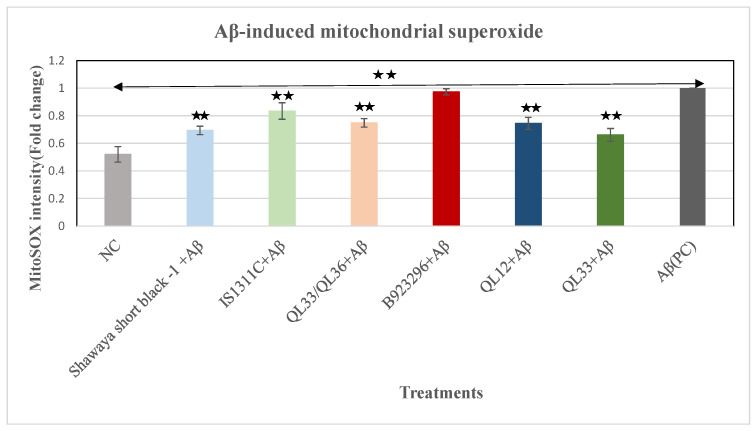
The neuroprotective effects of the PP-rich extracts from the six different varieties of sorghum on the Aβ-induced mitochondrial superoxide in the BE (2)-M17 cells. The relative MitoSOX fluorescence intensity was analyzed by the processing of images taken from the fluorescence microscopy. The mean ± SD of three independent experiments is presented. The statistically significant differences in comparison to the PC are marked **, where *p* ≤ 0.01. NC = negative control, PC = positive control.

To measure mitochondrial superoxide levels, MitoSOX^TM^ Red was used. As shown in Figure 5, adding Aβ to the cells significantly increased the mitochondrial superoxide level by 92.4% (*p* ≤ 0.01). The optimum dosage of the sorghum extracts of Shawaya short black-1, IS1311C, QL33/QL36, QL12, and QL33 significantly mitigated mitochondrial superoxide levels by 30.6%, 12.2%, 23.2%, 25.5%, and 33.9%, respectively (*p* ≤ 0.01), while the extract of B923296 had no significant effect.

### 3.11. Effect of PP-Rich Sorghum Extracts on TBHP-Induced Mitochondrial Superoxide in BE (2)-M17 Cells

**Figure 6:**
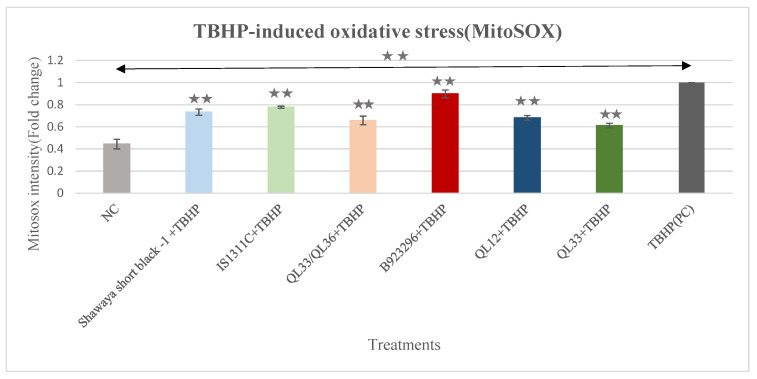
The neuroprotective effects of the PP-rich extracts from the six different varieties of sorghum on the TBHP-induced mitochondrial superoxide in the BE (2)-M17 cells. The relative MitoSOX fluorescence intensity was analyzed by the processing of images taken from fluorescence microscopy. The mean ± SD of three independent experiments is presented. The statistically significant differences in comparison to the PC are marked **, where *p* ≤ 0.01. NC = negative control, PC = positive control.

The MitoSOX assay kit was also used to measure the level of mitochondrial superoxide in the cells treated with TBHP plus the sorghum extracts. As shown in Figure 6, TBHP increased the mitochondrial superoxide level by 125% when compared to the negative control group (*p* ≤ 0.01).

All extracts demonstrated a significant attenuation (*p* ≤ 0.05) of mitochondrial superoxide. In summary, attenuation percentages were 26.7% for the optimum dosage of Shawaya short black -1, 22.3% for IS1311C, 34.3% for QL33/QL36, 10.2% for B923296, 31.9% for QL12, and 38.8% for QL33.

## 4. Discussion

Sorghum grains, depending on variety, contain a large number of different PPs, including the 3-deoxyanthocyanidins luteolinidin and apigeninidin that are very rare in nature [36]. The consumption of sorghum foods, due to these PPs, has the potential to decrease the risk of several chronic diseases due to their vasoprotective and anti-inflammatory activities [37]. These 3-deoxyanthocyanidins have high stability in light, heat, and change in pH [38]; thus, they survive food processing [3] and there is evidence that they are more bioavailable than other anthocyanins [39]. Of those PPs found in sorghum grains, several studies have demonstrated the anti-AD properties of isolated luteolin [40], apigenin [41], taxifolin [42], naringenin [43], trans-cinnamic acid [44], ferulic acid [45], and caffeic acid [46]. It is known that the biological activity of plant-based foods is affected not only by the total amount of phenolic compounds but also by the profile of the individual phenolic compounds [47]. Therefore, understanding both the total and individual phenolic compound content of sorghum is important for a better understating of the possible health-beneficial effects of including sorghum foods in the diet.

Despite the considerable content of phenolic compounds (PPs) in sorghum grain, particularly in certain colored varieties, research investigating its potential neuroprotective effects against conditions like Alzheimer’s disease (AD) remains limited [4]. There is growing interest in exploring the effects of phenolic compounds on AD due to their diverse biological activities [48]. These activities include inhibiting Aβ aggregation, chelating metals, preventing mitochondrial dysfunction and apoptosis, as well as possessing antioxidant and anti-inflammatory properties, all of which are suggested pathways for potential anti-AD effects [48]. Given that defective Aβ clearance is implicated in most AD patients, discovering methods to reduce Aβ aggregation or disaggregate preformed Aβ holds promise as a therapeutic strategy [49]. Phenolic compounds (PPs) need specific characteristics to effectively reduce Aβ fibril aggregation [50]. Research indicates that PPs containing an aromatic ring with two or more adjacent hydroxyl groups are more potent inhibitors of Aβ plaque and/or tau tangles. For instance, sorghum’s distinctive 3-deoxyanthocyanins, luteolinidin and apigeninidin, possess these structural features, suggesting their potential for protecting against Alzheimer’s disease (AD) [15,39].

Oxidative stress, either as a cause or consequence of Aβ and tau aggregation, significantly contributes to the neuropathogenesis of AD [51]. Aβ exerts its neurotoxicity through different pathways such as ROS production and neuronal death [52,53]; an imbalance between ROS production in the brain and antioxidant defense ultimately leads to oxidative damage and neuronal dysfunction [1,54]. Several PPs found in sorghum such as ferulic acid, caffeic acid, trans-resveratrol, quercetin, catechin, cinnamic acid, taxifolin, apigenin, and kaempferol are reported to be beneficial for the attenuation of cellular pathways contributing to AD, particularly oxidative stress pathways when studied as purified compounds [5,6,43,44,55,56]. The unique sorghum PPs 3DXAs have demonstrated high antioxidant and anti-inflammatory activities [39,57]. The sorghum varieties used in this study have also been reported to contain high levels of ferulic acid and caffeic acid which both have strong anti-inflammatory and free radical-scavenging abilities [45,58,59,60]. Ferulic acid has been shown to protect neurons against Aβ-induced oxidative stress in vitro [55]. Furthermore, studies have demonstrated that administering caffeic acid improves memory in the Aβ-induced mouse model of Alzheimer’s disease (AD) [46]. Additionally, the sorghum varieties investigated in this study contain other phenolic compounds renowned for their potent antioxidant and anti-inflammatory properties, such as luteolin and apigenin, which may contribute to their anti-AD activity [61,62,63]. It is also reported that the oral administration of apigenin and luteolin assisted the learning and memory impairment of Aβ-induced mice through several pathways including attenuating oxidative stress [61,62]. Additionally, some of the sorghum varieties herein are reported to contain taxifolin and naringenin [26]. Taxifolin and naringenin are both flavonoids that exhibit potent anti-inflammatory and antioxidant properties [64,65,66]. Taxifolin significantly attenuated Aβ-induced cognitive impairment and neuronal cell death in a mice model of AD [42]. Conversely, in vivo studies have indicated that naringenin attenuated learning and memory deficits by diminishing oxidative stress [43,56].

The present study provided phenolic compound (PP) contents for sorghum grain varieties that diverged from the previously reported values for the same varieties [26]. These variations were likely due to the choice of solvents. The previous study [26] employed methanol, resulting in higher extraction levels compared to the food-grade solvent (ethanol) utilized in our research. Particularly for Shawaya short black-1 and IS1311C, the differences were considerable, though not significant for the other varieties. Nevertheless, the ranking of PP contents among the varieties in our study aligned with the findings of Wu et al. [26].

Our cellular studies demonstrated that extracts from all six tested sorghum varieties provided significant protection against Aβ-induced toxicity at optimal doses. These optimal doses exhibited a more robust protective effect compared to lower doses (equivalent to half the optimal dose) across all varieties. The extracts may preserve cell viability from Aβ toxicity through three potential mechanisms: the deposition of less toxic Aβ plaques, inhibition of Aβ aggregation, or disaggregation of preformed Aβ aggregates [67]. Both the DMSO-dissolved and treatment-media-dissolved extracts of sorghum QL33 showed the highest protective capacity at the optimum dosage. However, the different levels of protection across sorghum varieties observed could arise from variations in extract concentrations used in the assay, which were determined by toxicity levels, and differences in the type and quantity of specific phenolic compounds (PPs) present in each extract. These differences might influence toxicity levels and the cellular bioavailability of the phenolic compound mixtures [68]. QL33, for example, was less toxic, which allowed us to use higher concentrations. In addition, the HPLC analysis revealed that QL33 had high levels of caffeic acid, ferulic acid, and apigenin, which were previously shown to be highly effective in attenuating Aβ toxicity [41,45,46]. Additionally, eight of the nine quantified PPs were identified in QL33 which in combination may have given higher protective activity through targeting multiple protective mechanisms.

This study showed that the DMSO PP extracts offered better protection against Aβ-induced cellular damage compared to the treatment media extracts (Figure 2). The increased protective effect might be attributed to the higher solubilizing ability of DMSO compared to the treatment media, potentially aiding in the interaction of the phenolic compounds (PPs) with the cells [69].

The Th-T results of this study not only show the ability of these PP-rich extracts to inhibit the Aβ42 aggregation process but also provide insight into the underlying mechanism of action for protecting cell viability against Aβ toxicity. The PP in the sorghum extracts may have bound with the monomer protein and reduced its aggregation through electrostatic repulsion of the PP-monomer complexes. The Th-T results demonstrate the efficacy of the PP-rich extracts from the six different sorghum varieties in inhibiting Aβ42 aggregation. The observed stronger inhibition activity of the sorghum Shawaya short black-1 compared to B923296 could be attributed to its higher level of proanthocyanidins, which have been previously reported to inhibit Aβ aggregation [15]. The reduced binding in the ThT in the presence of the sorghum extracts might also be a consequence of the direct binding of the PP to the already aggregated protein. Further studies using electron microscopy are warranted to investigate this phenomenon.

In vitro and animal studies have previously demonstrated the anti-Aβ and anti-AD activity of polyphenolic extracts that contain some of the PPs we have identified in the sorghum extracts. For example, Ouattara et al., [70] demonstrated that *Nelsonia canescens* extracts contain various polyphenolic compounds such as p-coumaric acid, caffeic acid, chlorogenic acid, ferulic acid, gentisic acid, apigenin, luteolin, and quercetin which exhibit anti-AD activity through antioxidant and acetylcholinesterase (AChE) inhibition pathways. Additionally, research indicates that the daily consumption of the polyphenolic extract of wine which includes ferulic acid, resveratrol, proanthocyanidins, and caffeic acid for 2 months resulted in decreased brain levels of both Aβ1-40 and Aβ1-42 of 3x Tg-AD mice [71].

In this study, oxidative stress was induced using TBHP, a well-established oxidative stress inducer, alongside Aβ. Aβ has been implicated in neurotoxicity through its impact on the mitochondria and the generation of reactive oxygen species (ROS) [6]. While Aβ is recognized as a primary contributor to oxidative damage in Alzheimer’s disease (AD), TBHP serves as a known ROS inducer, leading to lipid peroxidation, DNA oxidative damage, and an elevation in Aβ levels in cellular models [72]. Our findings demonstrate that optimized dosages of five out of the six extracts of the different sorghum varieties used in this study significantly reduced Aβ-induced ROS/MitoSOX in the range of 29.1% to 43.6% for Aβ-induced ROS and 12.2% to 33.9% for Aβ-induced MitoSOX. The most substantial decrease was observed for QL33, potentially attributed to its non-toxicity at higher concentrations, allowing for the use of a higher dose. Additionally, we evaluated the lower dosage of each extract as an added measure to gauge its effectiveness in attenuating ROS. As anticipated, the lower dosages (half of the optimal dosage) were less effective in attenuating ROS across all sorghum varieties. The efficacy of both the optimal and lower dosages varied depending on the specific variety and dosage administered. Oxidative stress was induced by TBHP, and our data revealed that the optimized dosages of the extracts from all six sorghum varieties significantly decreased ROS/MitoSOX levels by 24.1% to 52.3% for ROS and 10.2% to 38.8% for MitoSOX.

The present results align with a previous study indicating that sorghum extracts effectively reduced intracellular ROS levels [73]. Further, the study by Ajiboye et al. also showed the beneficial effect of the polyphenolic extracts of sorghum in the detoxification of ROS in N-nitrosodiethylamine-treated rats [74]. However, neither of these studies targeted specific AD-related pathologies.

Variations in effectiveness among the different sorghum varieties in the current work may be attributed to the toxicity of certain extracts, such as Shawaya short black-1 and IS1311C, at higher dosages, prompting the utilization of lower concentrations for these specific varieties. Additionally, the differences in their content, including the type and level of each phenolic compound, could be another contributing factor. However, attributing specific behavior and performance to the specific content is challenging due to the abundance of phenolic compounds [26]. Moreover, the combination of individual phenolic compounds in their natural state may exert a completely different protective mechanism compared to each compound individually.

We have also investigated the non-cellular antioxidant activity of the sorghum extracts through the ABTS and DPPH assays. The sorghum varieties Shawaya short black -1 and IS1311C showed the highest level of antioxidant activity which agrees with a study by Wu. et al. [26]. A detailed review comparing chemical and cellular antioxidant tests revealed that the antioxidant strength measured by chemical tests does not directly reflect effectiveness in cells or living organisms, despite being useful for initial assessments. This is because true in vivo antioxidant activity involves various factors such as increasing antioxidant enzymes, altering cell signals, absorption differences among sorghum types, and gene activity [68]. Hence, it is typically challenging to forecast how cells will respond to treatment solely based on chemical assay results. Transitioning to cellular assays becomes crucial for a more accurate evaluation of extract performance.

The DCFDA has been widely used to test the hydroxyl, peroxyl, and ROS molecules within cells due to its rapid, high-sensitivity, and simple measurement methods. This cell fluorescein-based indicator diffuses into cells as a colorless, non-fluorescent probe until the two acetate groups are cleaved by intracellular esterase to yield the fluorescent fluorophore, 5-(and-6)-carboxy-2′,7′-dichlorofluorescein [75]. In contrast, MitoSOX is a fluorogenic dye that targets mitochondrial superoxide (mitochondrial superoxide is the main source of cellular oxidation). MitoSOX is produced by conjugating triphenyl phosphonium ion (TPP^+^) to combine different molecules into the highly electronegative mitochondrial matrix to dihydroethidium [76]. Dihydroethidium creates the superoxide-specific oxidation by-product called 2-hydroxyethidium if oxidated by superoxide; if not, it creates other by-products when oxidized by ROS other than superoxide [76,77].

In summary, this study adds valuable evidence to the potential of sorghum polyphenols for AD risk reduction as recently reviewed [75] since it marks the first demonstration that phenolic compound-rich extracts from sorghum grains provide significant attenuation effects by reducing Aβ-induced cell death and diminishing Aβ aggregation. These effects were dependent on the sorghum variety and dose of the extract. The sorghum extracts were shown to attenuate cellular oxidative stress in a dose- and variety-dependent manner through the antioxidant pathway. We observed a possible neuroprotective effect of sorghum polyphenolic extracts by attenuating ROS in general and targeting the mitochondria in particular. The sorghum varieties, particularly Shawaya short black-1 and IS1311C, demonstrated high levels of total phenolics, flavonoids, and antioxidant capacity. The varieties exhibited distinct polyphenol profiles. The extracts from all the varieties improved cell viability compared to the control group. QL33 showed the strongest protection against Aβ-toxicity cell death. All the sorghum extracts reduced Aβ aggregation to varying extents. Additionally, except for variety B923296, all the extracts significantly decreased ROS and mitochondrial superoxide levels induced by Aβ and TBHP compared to the control, with effects varying by dosage and variety. These data provide promising evidence for the potential AD-protective effects of the sorghum PP. To strengthen the evidence of these extracts’ impact on Aβ42 aggregation, additional complementary assays such as Western immunoblot analysis should be conducted to detect the formation of oligomers. Furthermore, further investigations are warranted to verify the influence of phenolic compounds on tau and inflammatory pathways, as well as their neuroprotective properties and bioavailability. These studies will provide a more comprehensive understanding of the potential benefits and mechanisms underlying the observed effects of the phenolic compound-rich extracts from sorghum grains. The results from this study indicate the potential value of the incorporation of high PP sorghum in the diet. There is currently an increase in the use of colored grain sorghum varieties for human foods giving consumers greater opportunity to consume these. Likewise, nutraceutical extracts in the form of dried extract powder in capsules could be commercially manufactured from sorghum grain.

## Figures and Tables

**Table 1:** Polyphenolic (mg GAE/g grain, db) and flavonoid levels (mg CE/100 g, grain, db) of six sorghum varieties.

	Varieties					
	Shawaya Short Black-1	IS1311C	QL33/QL36	B923296	QL12	QL33
** Phenolics **	7.14 ± 0.67 ^c^	4.77 ± 0.41 ^b^	1.59 ± 0.23 ^a^	1.97 ± 0.57 ^a^	1.62 ± 0.25 ^a^	2.13 ± 0.36 ^a^
** Flavonoids **	5.24 ± 0.01 ^d^	3.3 ± 0.4 ^c^	1.28 ± 0.08 ^b^	0.81 ± 0.05 ^a^	0.83 ± 0.01 ^a^	1.19 ± 0.14 ^a^

Values with different superscripts in the same row are significantly different (*p* ≤ 0.05). GAE: gallic acid equivalents. CE: catechin equivalents. db: dry basis. Mean ± SD (*n* = 4).

**Table 2:** Content of individual polyphenols (µg/g grain db) from six sorghum varieties.

	Variety					
	Shawaya Short Black-1	IS1311C	QL33/QL36	B923296	QL12	QL33
** Luteolinidin **	38.0 ± 7.4 ^d^	3.2 ± 0.7 ^a^	6.3 ± 0.3 ^b^	Nd	8.7 ± 0.4 ^c^	6.6 ± 0.2 ^b^
** Apigeninidin **	120.5 ± 17.7 ^e^	3.8 ± 0.4 ^b^	28.9 ± 3.0 ^d^	8.0 ± 2.30 ^c^	1.4 ± 0.4 ^a^	7.2 ± 0.9 ^c^
** Luteolin **	12.6 ± 1.4	nd	nd	nd	nd	nd
** Apigenin **	9.0 ± 2.2 ^a^	14.2 ± 0.1 ^b^	Nd	17.8 ± 0.59 ^c^	nd	38.6 ± 0.3 ^d^
** Taxifolin **	15.2 ± 0.5 ^a^	16.2 ± 0.2 ^a^	nd	nd	nd	nd
** Naringenin **	11.9 ± 2.3 ^c^	10.7 ± 0.1 ^c^	6.2 ± 0.10 ^b^	5.3 ± 0.07 ^a^	nd	6.6 ± 0.3 ^b^
Cinnamic acid *	6.0 ± 0.1 ^a^	nd	5.7 ± 0.08 ^a^	nd	6.1 ± 0.3 ^a^	6.1 ± 0.3 ^a^
** Ferulic acid **	15.8 ± 1.7 ^c^	28.3 ± 0.2 ^e^	4.8 ± 0.15 ^a^	19.9 ± 0.40 ^d^	6.4 ± 0.3 ^b^	55.8 ± 0.5 ^f^
** Caffeic acid **	38.9 ± 2.9 ^b^	32.1 ± 0.0 ^b^	35.2 ± 0.48 ^b^	30.7 ± 0.30 ^a^	33.16 ± 1.4 ^b^	34.7 ± 0.3 ^b^

Values with different superscripts in the same row are significantly different (*p* ≤ 0.05). db = dry basis. nd = not detected. Mean ± SD (*n* = 4). * trans form.

**Table 3:** Antioxidant capacity of six varieties of sorghum whole grains (mg TE/g original grain, dry basis).

	Variety					
	Shawaya Short Black -1	IS1311C	QL33/QL36	B923296	QL12	QL33
** DPPH **	20.36 ± 0.152 ^d^	22.09 ± 0.04 ^e^	1.12 ± 0.02 ^b^	0.65 ± 0.04 ^a^	0.65 ± 0.00 ^a^	1.52 ± 0.02 ^c^
** ABTS^+^**	45.01 ± 0.216 ^e^	45.35 ± 0.14 ^f^	3.04 ± 0.14 ^b^	3.65 ± 0.00 ^c^	2.64 ± 0.00 ^a^	4.24 ± 0.04 ^d^

Mean ± SD; Values with different superscripts in the same row are significantly different (*p* ≤ 0.05); TE: Trolox equivalents.

## Data Availability

The original contributions presented in the study are included in the article, further inquiries can be directed to the corresponding author.
